# T237. USER EXPERIENCE TOWARDS AN INTEGRAL INTERVENTION MODEL BASED ON M-HEALTH SOLUTION FOR PATIENTS WITH TREATMENT-RESISTANT SCHIZOPHRENIA (M-RESIST): QUALITATIVE INFORMATION FROM PATIENTS, CAREGIVERS AND CLINICIANS IN A PILOT STUDY

**DOI:** 10.1093/schbul/sby016.513

**Published:** 2018-04-01

**Authors:** Silvia Marcó-García, Elena Huerta-Ramos, Susana Ochoa, Eva Grasa, Anna Alonso, Erika Jääskeläinen, Elisenda Reixach, Jesús Berdún, Charlotte Jewel, m-RESIST GROUP, Iluminada Corripio, Judith Usall

**Affiliations:** 1 Parc Sanitari Sant Joan de Deu; 2 IIB-Hospital Santa Creu I Sant Pau; 3 Hospital Sant Pau; 4 University of Oulu; 5 Fundació TicSalut; 6 IMEC; 7 Hospital Santa Creu i St. Pau, CIBERSAM; 8 Parc Sanitari San Joan de Dèu, CIBERSAM

## Abstract

**Background:**

In the European Union approximately 5 million people suffer from psychotic disorders. Patients with schizophrenia make up the largest subgroup of these, and between 30–50% of them are considered resistant to treatment. Despite the proven potential of m-health solutions, there remains a lack of technological solutions in the treatment of patients with this disease. To improve the quality of care of these outpatients, an m-health solution termed Mobile Therapeutic Attention for Patients with Treatment Resistant Schizophrenia (m-RESIST) has been created in European Union and implemented in three countries (Spain, Hungary and Israel). m-RESIST is an innovative project aimed to empower patients with Treatment Resistant Schizophrenia, which integrates pharmacological and psychosocial approaches, develops knowledge of the illness using predictive models, and includes the following m-Health tools: a Dashboard, a Smartwatch and a Smartphone. Prior to the implementation in the healthcare reality, the solution has been tested in pilot groups to assess the acceptability, usability and satisfaction of all m-RESIST components in each country. In addition to online and onsite visits, this phase has included an anonymous online questionnaire, with the aim of capturing more consistently the opinion of participants in their experience with m-RESIST. We summarize their opinions about services and devices included in the solution, as well as the improvement proposals of each group.

**Methods:**

During three months (from August to October), a case manager from Spain sent out an interval question to the Spanish participants via m-RESIST Dashboard, in order to collect information about the users experience with the system. It was administered weekly on different days and at different times, being anonymous for both parts. We have obtained qualitative information from nine patients, one caregiver and two clinicians.

**Results:**

Patients consider m-RESIST a useful tool, in terms of immediacy of contact with clinicians, improvement of disease awareness, better follow-up of their disease, less-worries from caregivers and feeling protected by having a team with whom they can share their concerns. As cons, patients have a strong feeling of being observed and with too much repetitive questionnaires to answer. They consider a bit difficult to use the devices, with several errors in its operation. They do not like to carrying the smartwatch and to check the battery of the devices. Also, the program is not available on weekends, which leads to a feeling of being somehow disregarded. For patients, this solution should also include the possibility of changing programmed location when on vacations and it should not be a substitute for traditional treatment. Regarding caregivers, m-RESIST is considered as a good tool to have in their daily lives, because it helps in terms of disease improvement, to have a better follow-up about pharmacological issues and symptoms, and to feel secure knowing there is a support for both patient and caregiver. No cons were reported. For clinicians, m-RESIST is a system with high potential, being easy, intuitive and useful, specially to share psychoeducational content with patients and to improve communication with them. However, several technological problems must be solved in the future, there still provide a poor patient monitoring and much more time is needed than regarding the traditional treatment.

**Discussion:**

The three user groups consider m-RESIST as a useful tool, with pros and cons being described regarding their specific needs and provided proposals for improvement.

